# An interpreting machine learning models to predict amputation risk in patients with diabetic foot ulcers: a multi-center study

**DOI:** 10.3389/fendo.2025.1526098

**Published:** 2025-03-25

**Authors:** Haoran Tao, Lili You, Yuhan Huang, Yunxiang Chen, Li Yan, Dan Liu, Shan Xiao, Bichai Yuan, Meng Ren

**Affiliations:** ^1^ Department of Endocrinology, Sun Yat-Sen Memorial Hospital, Sun Yat-sen University, Guangzhou, China; ^2^ Guangdong Clinical Research Center for Metabolic Diseases, Guangzhou Key Laboratory for Metabolic Diseases, Guangzhou, China; ^3^ Department of Endocrinology, Shantou Central Hospital, Shantou, China; ^4^ Department of Endocrinology, Dongguan People’s Hospital Puji Branch, Dongguan, China; ^5^ Department of Endocrinology, People’s Hospital of Shenzhen Baoan District, Second Affiliated Hospital of Shenzhen University, Shenzhen, China; ^6^ Department of Endocrinology, Jieyang People’s Hospital, Jieyang, China

**Keywords:** diabetic foot ulcer, lower extremity amputation, risk factor, machine learning, SHAP

## Abstract

**Background:**

Diabetic foot ulcers (DFUs) constitute a significant complication among individuals with diabetes and serve as a primary cause of nontraumatic lower-extremity amputation (LEA) within this population. We aimed to develop machine learning (ML) models to predict the risk of LEA in DFU patients and used SHapley additive explanations (SHAPs) to interpret the model.

**Methods:**

In this retrospective study, data from 1,035 patients with DFUs at Sun Yat-sen Memorial Hospital were utilized as the training cohort to develop the ML models. Data from 297 patients across multiple tertiary centers were used for external validation. We then used least absolute shrinkage and selection operator analysis to identify predictors of amputation. We developed five ML models [logistic regression (LR), support vector machine (SVM), random forest (RF), k-nearest neighbors (KNN) and extreme gradient boosting (XGBoost)] to predict LEA in DFU patients. The performance of these models was evaluated using several metrics, including the area under the receiver operating characteristic curve (AUC), decision curve analysis (DCA), precision, recall, accuracy, and F1 score. Finally, the SHAP method was used to ascertain the significance of the features and to interpret the model.

**Results:**

In the final cohort comprising 1332 individuals, 600 patients underwent amputation. Following hyperparameter optimization, the XGBoost model achieved the best amputation prediction performance with an accuracy of 0.94, a precision of 0.96, an F1 score of 0.94 and an AUC of 0.93 for the internal validation set on the basis of the 17 features. For the external validation set, the model attained an accuracy of 0.78, a precision of 0.93, an F1 score of 0.78, and an AUC of 0.83. Through SHAP analysis, we identified white blood cell counts, lymphocyte counts, and blood urea nitrogen levels as the model’s main predictors.

**Conclusion:**

The XGBoost algorithm-based prediction model can be used to dynamically estimate the risk of LEA in DFU patients, making it a valuable tool for preventing the progression of DFUs to amputation.

## Introduction

At present, more than 550 million people are diagnosed with type 2 diabetes mellitus (T2DM) globally, and the prevalence of T2DM continues to increase (1). Projections indicate that by 2045, the number of individuals diagnosed with diabetes worldwide will increase to 700 million (1). Moreover, advancements in medical treatment have substantially prolonged the life expectancy of individuals with diabetes, leading to a notable increase in the prevalence of chronic diabetic complications (2). Among the myriad of diabetic complications, diabetic foot ulcers (DFUs) constitute a particularly severe and prevalent issue. DFUs are distinguished not only by their notably high mortality rate but also by their substantial contribution to approximately 85% of nontraumatic amputations worldwide (3). A previous study indicated that patients with DFUs perceive the risk of lower-extremity amputation (LEA) as a more significant concern than mortality throughout the progression of the disease (4). This phenomenon is attributable to the significant effects of LEA on patients’ physical and psychological well-being, leading to prolonged hospitalization, considerable financial strain, intricate treatment protocols, and a significantly diminished quality of life. Moreover, patients with DFUs who have undergone LEA have a poor prognosis, with a 3-year mortality rate of 35–50% (5) and a 5-year mortality rate of 52–80% (6). Consequently, conducting personalized assessments for patients with DFUs to evaluate their risk of amputation and identify associated risk factors can provide essential insights for early intervention treatments. The results of this analysis are expected to be useful for reducing the incidence of amputation surgeries, decreasing patient mortality rates, and lowering healthcare costs.

Presently, widely utilized classification systems for DFU, such as the Diabetic Ulcer Severity Score, the Meggitt–Wagner classification, and the University of Texas Diabetic Wound Classification, serve as standard instruments for informing treatment strategies and predicting the risk of disease progression in patients with DFUs (7–9). Although these classification systems have the potential to predict amputation risk in patients, they have not been universally adopted as the gold standard (10). This limitation stems primarily from practitioners’ reliance on clinical experience rather than objective statistical data for scoring. Furthermore, these systems cannot be used to integrate demographic information, clinical and laboratory data, medical history, foot condition, and other pertinent risk factors comprehensively (11, 12). This limitation has led to diminished sensitivity and specificity in predicting amputation risk among patients with DFUs.

DFUs present substantial complexity owing to the clinical heterogeneity observed among patients and the multimodal data obtained from various disciplines, such as imaging, surgery, and endocrinology. To elucidate the complexity of DFUs, it is imperative to use advanced analytical methodologies, such as machine learning (ML) and artificial intelligence (AI) (13). These data analysis techniques are used to develop algorithms for predicting outcomes by “learning” from data (14). Through the utilization of ML, clinical physicians can now predict the healing trajectories of DFUs, assess the risk of amputation, and develop personalized treatment plans on the basis of clinical data. Several studies have investigated the application of ML techniques in predicting diabetic foot amputations (15). However, the sample sizes in some studies are relatively small, which may limit their representativeness of the population (16–18).Moreover, several of these small-sample studies employ only a single type of ML algorithm for model development (18, 19). Consequently, there is an urgent need for the development of more sophisticated and advanced models capable of effectively addressing the heterogeneity observed in patients with DFUs. While various ML algorithms have been employed in several studies, their complex nature might limit their interpretation by patients and clinicians in real-world clinical settings (16, 17). The “black-box” nature of traditional ML algorithms poses challenges in explaining the specific patient characteristics that contribute to a particular prediction. The limited interpretability of ML methods constrains their application in medical decision support and it is also one of the significant barriers to their implementation in real-world clinical settings (20). To overcome these limitations, our study incorporated the ML algorithm with SHapley Additive exPlanations (SHAPs) (21). In addition to enhancing the precision of amputation risk prediction in patients with DFUs, SHAP provides intuitive explanations that empower patients to understand their own risk factors. It can aid clinicians in comprehending the decision-making process for evaluating disease severity and optimizing opportunities for early intervention, while also contributing to the development of interpretable and personalized risk prediction models.

In short, the small sample sizes of amputee patients, coupled with the limited interpretability of models, constrains the application of priors ML models in medical decision support systems. Thus, this study aimed to utilize data from multiple medical centers concerning patients with DFUs to develop and evaluate various ML models, ultimately identifying the best model for predicting the risk of amputation during hospitalization. Furthermore, SHAPs were used to visualize the optimal model and investigate the factors influencing the prognosis of DFUs. The objective was to equip healthcare providers with a concise and valuable instrument for identifying DFU patients at risk of amputation, enabling effective interventions to optimize clinical outcomes and improve the quality of life for these patients.

## Methods

**Figure 1:**
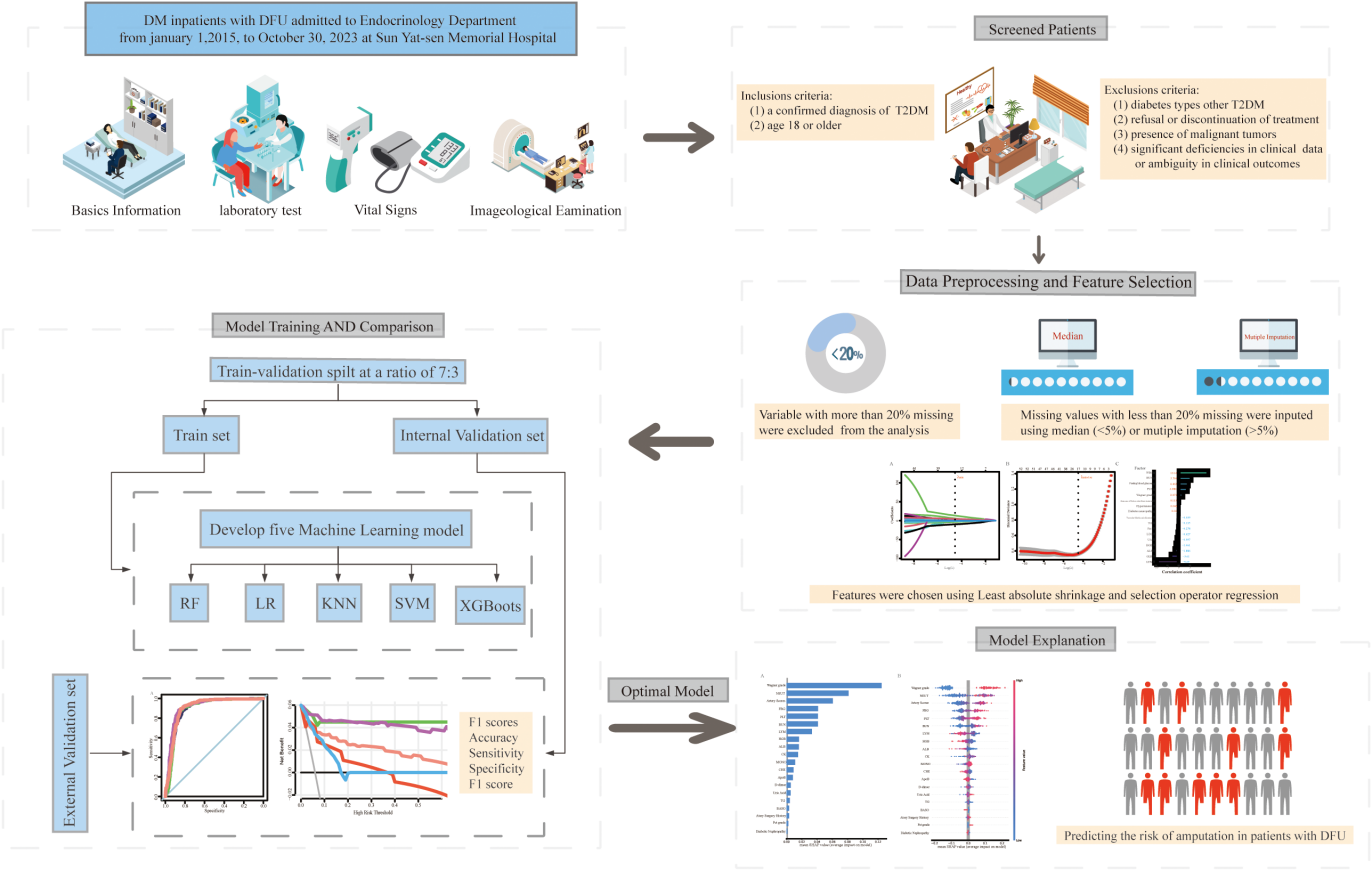
Workflow for constructing explainable machine learning models for predicting the risk of amputation in diabetic foot ulcer patients. DFU, diabetic foot ulcer; T2DM, type 2 diabetes mellitus; KNN, k-nearest neighbors; LR, logistic regression; SVM, support vector machine; RF, random forest; XGBoost, extreme gradient boosting.

In this retrospective cohort study, we developed a series of ML models to predict the risk of amputation in patients with DFUs. This study involved the development, validation and subsequent interpretation of the models. Following the preprocessing of the data and the selection of relevant variables, models were constructed utilizing 5 distinct ML algorithms. The model performances were subsequently evaluated via both internal and external validation datasets to identify the optimal model. Finally, SHAP was used to elucidate the optimal model. **Figure 1** illustrates the comprehensive research process, including the criteria for inclusion and exclusion, data preprocessing, feature selection, dataset partitioning, model development and validation, model comparison, and selection and interpretation of the optimal model.

### Study population and outcome

Data for the training cohort were derived from the clinical records of patients with DFUs (Wagner grades 1–5) (22) admitted to the Endocrinology Department at Sun Yat-sen Memorial Hospital from January 2015 to October 2023. The inclusion criteria were as follows: (1) a confirmed diagnosis of T2DM and (2) over the age of 18. The exclusion criteria included: (1) diabetes types other than T2DM; (2) refusal or discontinuation of treatment; (3) the presence of malignant tumors; and (4) significant deficiencies in clinical examination data or ambiguity in clinical outcomes. On the basis of these criteria, a total of 1035 T2DM patients with DFUs were included in the training cohort.

With data collected between January 2020 and October 2023, the external validation cohort consisted of patients with T2DM and DFUs from Shantou Central Hospital, Dongguan People’s Hospital, Jieyang People’s Hospital and Shenzhen Central Hospital. The inclusion and exclusion criteria were consistent with those of the training cohort. Finally, the external validation cohort included a total of 297 patients with T2DM complicated by DFUs. This study received approval from the Ethics Committee of Sun Yat-sen Memorial Hospital. Given that all patient data were anonymized and that the study did not influence clinical decision-making, the requirements for individual patient consent and an ethical informed consent statement were waived. The baseline characteristics of the demographic and clinical variables for the training cohort and external validation cohorts are detailed in **Table 1** and **Supplementary Table 1**.

**Table 1:** Characteristics of the training cohort patients at first admission.

Factors	Total (n=1035)	Non-amputation (n=563)	Amputation (n=472)	*P* values
Demographics				
Age, yr	66.2 ± 12.2	64.6 ± 12.6	68.2 ± 11.5	<0.001
Duration of diabetes, yr	10.0 (5.0-20.0)	10.0 (5.0-20.0)	11.0 (5.0-20.0)	0.465
Sex, n (%)				0.210
Male	631 (61.0%)	341 (60.6%)	290 (63.0%)	
Female	404 (39%)	222 (39.4%)	182 (37.0%)	
BMI, kg/m (2)	23.2 ± 3.7	23.7 ± 3.9	22.6 ± 3.3	<0.001
SBP, mmHg	135.5 ± 22.8	136.1 ± 22.6	134.6 ± 23.0	0.281
DBP, mmHg	72.9 ± 11.3	73.4 ± 11.4	72.3 ± 11.2	0.120
Medical history				
Hypertension, n (%)	324 (31.3%)	155 (27.5%)	169 (35.8%)	0.004
Smoking (current or ever), n (%)	324 (31.3%)	155 (27.5%)	169 (35.8%)	0.004
Drinking (current or ever), n (%)	125 (12.1%)	54 (9.6%)	71 (15%)	0.007
CAD, n (%)	161 (15.6%)	79 (14.0%)	82 (17.4%)	0.140
Cerebral infarction, n (%)	114 (11.0%)	50 (8.9%)	64 (13.6%)	0.017
PAD, n (%)	996 (96.2%)	532 (94.5%)	464 (98.3%)	0.03
Prior ulcer, n (%)	107 (10.3%)	43 (7.6%)	64 (16.3%)	0.002
Diabetic nephropathy, n (%)	511 (49.4%)	261 (46.4%)	250 (53.0%)	0.034
Diabetic peripheral neuropathy, n (%)	757 (73.1%)	400 (71.0%)	357 (75.6%)	0.097
Wagner classification system, n (%)				<0.001
I-III	520 (50.2%)	477 (84.7%)	43 (9.1%)	
IV-V	515 (49.8%)	86 (15.3%)	429 (90.9%)	
Clinical and laboratory data				
WBC count, ×10^9/^L	10.35 ± 5.62	7.2 ± 2.0	14.2 ± 6.1	<0.001
Hemoglobin, g/L	100.48 ± 25.96	114.2 ± 21.2	84.1 ± .21.1	<0.001
PLT count, ×10^9^/L	307 (207-407)	255 (207-315)	399.0 (312-472)	<0.001
LYM count, ×10^9^/L	1.64 ± 0.82	2.06 ± 0.79	1.14 ± 0.51	<0.001
NEUT count, ×10^9^/L	7.83 ± 5.50	4.64 ± 1.74	11.64 ± 6.0	<0.001
Potassium, mmol/L	4.09 ± 0.38	4.10 ± 0.38	4.08 ± 0.37	0.417
Sodium, mmol/L	140.48 ± 3.74	140.42 ± 3.26	140.56 ± 4.24	0.524
Phosphorus, mmol/L	1.15 ± 0.29	1.15 ± 0.26	1.16 ± 0.32	0.441
Calcium, mmol/L	2.10 ± 0.33	2.28 ± 0.14	1.89 ± 0.36	<0.001
Corrected calcium, mmol/L	2.29 ± 0.25	2.39 ± 0.11	2.18 ± 0.31	<0.001
Lactate dehydrogenase, U/L	191 (165-230)	185 (159-217)	203 (172-240)	<0.001
NT-proBNP, pg/ml	500.0 (143-1841)	294.0 (106-1024)	824.0 (252-3179)	<0.001
ALT, U/L	16 (11-24)	16 (11-24.)	16 (10-23.)	0.572
AST, U/L	19.0 (15-25)	19 (15-25)	19 (15-25)	0.486
Total bilirubin, μmol/L	8.30 (6.70-10.40)	8.00 (6.40-10.30)	8.50 (7.00-10.60)	0.059
Globulin, g/L	34.45 ± 6.60	36.52 ± 6.37	31.97 ± 5.99	<0.001
ALB, g/L	30.34 ± 7.28	34.46 ± 5.61	25.42 ± 5.85	<0.001
INR	1.11 ± 0.51	1.02 ± 0.18	1.21 ± 0.72	<0.001
APTT, s	29.17 ± 6.93	26.92 ± 4.03	31.86 ± 8.53	<0.001
Fibrinogen, g/L	5.16 ± 1.63	4.52 ± 1.34	5.92 ± 1.62	<0.001
D-dimer, mg/L	0.96 (0.52-1.90)	0.69 (0.40-1.14)	1.53 (0.79-2.92)	<0.001
BUN, mmol/L	8.23 ± 6.28	5.61 ± 3.31	11.36 ± 7.44	<0.001
Uric acid, μmol/L	317 (238-404)	301.0 (222.0-388.0)	333.00 (253.0-414.0)	<0.001
eGFR (ml·min-1·1.73 m-^2^)				<0.001
≤60, n (%)	399 (38.6%)	147 (26.1%)	252 (53.4%)	
>60, n (%)	636 (61.4%)	416 (73.9%)	220 (46.6%)	
Triglycerides, mmol/L	1.32 ± 0.86	1.49 ± 1.05	1.12 ± 0.48	<0.001
Total cholesterol, mmol/L	3.86 ± 1.31	4.24 ± 1.33	3.42 ± 1.14	<0.001
HDL, mmol/L	0.88 ± 0.29	0.96 ± 0.29	0.79 ± 0.25	<0.001
LDL, mmol/L	2.43 ± 0.92	2.69 ± 0.96	2.11 ± 0.76	<0.001
Fasting blood glucose, mmol/L	11.76 ± 5.21	8.96 ± 3.02	15.08 ± 5.33	<0.001
HbA1c, %	8.61 ± 2.35	8.39 ± 2.29	8.88 ± 2.40	0.01
Procalcitonin, ng/ml				<0.001
≤0.5, n (%)	889 (85.9%)	511 (90.8%)	378 (80.1%)	
>0.5, n (%)	146 (14.1%)	52 (9.2%)	94 (19.9%)	
Lower-limb vascular imaging examination				
Plaque, n (%)	974 (94.1%)	516 (91.7%)	458 (97.0%)	0.002
Vascular intima calcification, n (%)	169 (16.3%)	92 (16.4%)	77 (16.3%)	0.990
Vascular media calcification, n (%)	407 (39.3%)	222 (39.4%)	185 (39.1%)	0.938
Narrowing of below-the-knee arteries, n (%)	797 (77.0%)	399 (70.9%)	398 (84.3%)	<0.001
Stenosis of below-the-knee arteries, n (%)	474 (45.8%)	212 (37.7%)	262 (55.5%)	<0.001
Narrowing of above-the-knee arteries, n (%)	531 (51.3%)	260 (46.2%)	271 (57.4%)	<0.001
Stenosis of above-the-knee arteries, n (%)	121 (11.7%)	49 (8.7%)	72 (15.3%)	0.001

The mean ± standard deviation (SD) was calculated for continuous variables with normal distributions, and the p value was calculated via independent-samples t tests. Nonnormally distributed variables are expressed as medians (interquartile ranges), and comparisons were conducted via the Kruskal–Wallis test. Categorical data are presented as frequencies (percentages).

BMI, body mass index; SBP, systolic blood pressure; DBP, diastolic blood pressure; CAD, coronary artery disease; PAD, peripheral arterial disease; WBC, white blood cell; PLT, platelet; LYM, lymphocyte; NEUT, neutrophil; NT-proBNP, N-terminal pro b-type natriuretic peptide; ALT, alanine aminotransferase; AST, aspartate aminotransferase; GLB, globulin; ALB, albumin; INR, international normalized ratio; APTT, activated partial thromboplastin time; BUN, blood urea nitrogen; eGFR, estimated glomerular filtration rate; HDL, high-density lipoprotein; LDL, low-density lipoprotein.

The outcome of our study was amputation, which included both minor and major amputations (any LEA). The term major amputation refers to amputations above the ankle, and minor amputation refers to any amputation below the ankle.

### Study variables

Drawing upon contemporary research and clinical guidelines, we selected 62 potential predictive factors that may influence the risk of lower-limb amputation in patients with DF. The variables selected for this study included the following demographic and clinical characteristics: age, weight, height, body mass index (BMI), Wagner grade, smoking history, alcohol consumption history, and history of previous ulcers. Four comorbidities were considered: hypertension, history of cardiovascular disease, diabetic nephropathy (DN), and diabetic peripheral neuropathy (PND). Additionally, in the present study, we incorporated lower-limb vascular imaging examinations, which were conducted to assess vascular occlusion, vascular calcification, and arteriosclerosis. Furthermore, a total of 39 laboratory indicators were selected for the study, including D-dimer, C-reactive protein (CRP), the neutrophil count, hemoglobin (Hb), glycated Hb (HbA1c), triglycerides (TGs), low-density lipoprotein (LDL), albumin (ALB), blood urea nitrogen (BUN), and creatinine (Cr), among others.

### Data preprocessing and feature selection

Following the selection of study variables, data were extracted from the health information systems (HISs) of various hospitals. Indicators with more than 20% missing data were subsequently excluded from the analysis. Finally, a total of 55 candidate variables were selected within the training cohort. For variables with missing values constituting less than 5% of the data, imputation was performed using the median value. In cases where the proportion of missing data exceeded 5%, multiple imputation via random forest (RF) methodology was applied to address the missing values (23). To identify the most predictive factors for amputation and reduce the possibility of overfitting among the included variables, we used least absolute shrinkage and selection operator (LASSO) regression on the entire dataset of the training cohort. This approach facilitated the elimination of confounding variables, thereby enhancing model performance and mitigating the risk of overfitting. In LASSO regression, the coefficient estimates were regularized towards zero, with the degree of shrinkage being governed by an additional parameter, denoted as λ. To calculate the best possible values for λ, we used 10-fold cross-validation and iteratively applied LASSO regression to each fold. We subsequently identified the optimal tuning parameter (min λ) for the model by minimizing the cross-validation error and validated the model selection parameters to select the optimal predictive variable (24). After a thorough evaluation of the model features and their performance, we identified the min λ-1se as the final parameter for the Lasso model.

### ML models

As mentioned above, we sequentially undertook the development, validation, interpretation, and application of ML models in the current study. Initially, the patients in the training cohort were randomly partitioned, with 70% allocated to the training set and 30% to the internal validation set. We used five ML algorithms [extreme gradient boosting (XGBoost), support vector machine (SVM), RF, k-nearest neighbors (KNN) and logistic regression (LR)] to develop predictive models (25). In the training set, model hyperparameters were optimized to reduce overfitting and improve accuracy via GridSearch with tenfold cross-validation. We subsequently constructed receiver operating characteristic (ROC) curves and decision curve analysis (DCA) curves via the internal validation set. We then assessed the predictive performance of various ML models by calculating the accuracy, area under the curve (AUC), recall rate, precision rate, and F1 score (26, 27). The performance of the five models was further validated using an external dataset, employing the same methodological approach. We evaluated model performance via AUCs and F1 scores as the principal metrics and selected the optimal model on the basis of these metrics. To improve the interpretability of the ML model outcomes and analyze the contributing factors, we used SHAP to evaluate the feature importance of the optimal model.

### Statistical analysis

The normality of continuous variables was assessed via the Kolmogorov–Smirnov (K–S) test. Continuous variables with normal distributions are reported as the means (standard deviations, SDs) and were compared via independent samples t tests. Conversely, variables that did not follow a normal distribution are presented as the median (interquartile range) and were compared via the Kruskal–Wallis test. Categorical variables are presented as frequencies and percentages (n, %). Comparative analyses between the amputee and nonamputee groups were conducted via Student’s t test, the Mann–Whitney U test, or the chi–square test, contingent upon the distribution of the variables. A p value of less than 0.05 was considered to indicate statistical significance. Data preprocessing, model construction, validation, and interpretation of the ML models were executed via R Studio version 4.2 and Python (v. 3.8.3).

## Results

### Basic characteristics

**Figure 2:**
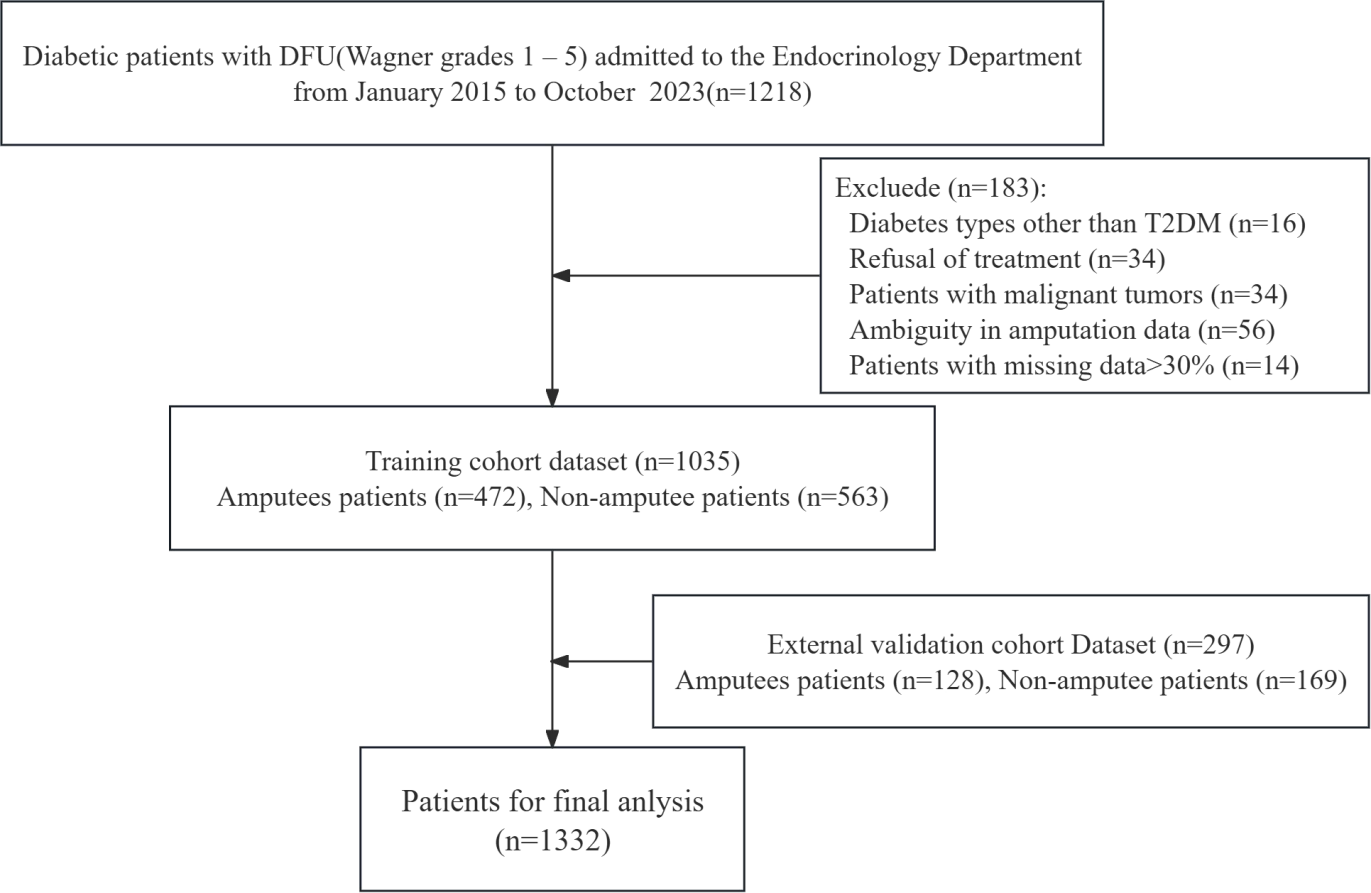
Flowchart of patient selection. T2DM, type 2 diabetes mellitus; DFU, diabetic foot ulcer.

As depicted in **Figure 2**, our study included a total of 1,332 patients diagnosed with DFUs. Among these, 1,035 patients were allocated to the training cohort, whereas 297 patients were assigned to the external validation cohort. The patients were categorized into two distinct groups on the basis of their posttreatment amputation status. In the training cohort, 45.6% (472/1035) of the patients underwent amputation, whereas in the external validation cohort, the incidence of amputation was 43.1% (128/297). Our data suggest that patients who underwent amputation presented elevated levels of inflammatory markers, including WBC and PCT, alongside increased serum Cr and BUN levels. Furthermore, these patients had increased levels of platelets (PLTs), D-dimers, and fibrinogen. Conversely, the levels of Hb, serum ALB, serum globulin (GLB), and lipids (including TGs, HDL, and LDL) were significantly lower in amputee patients than in nonamputee patients. The baseline characteristics of all the candidate variables for the training cohort are detailed in **Table 1**.

### Features selected

**Figure 3:**
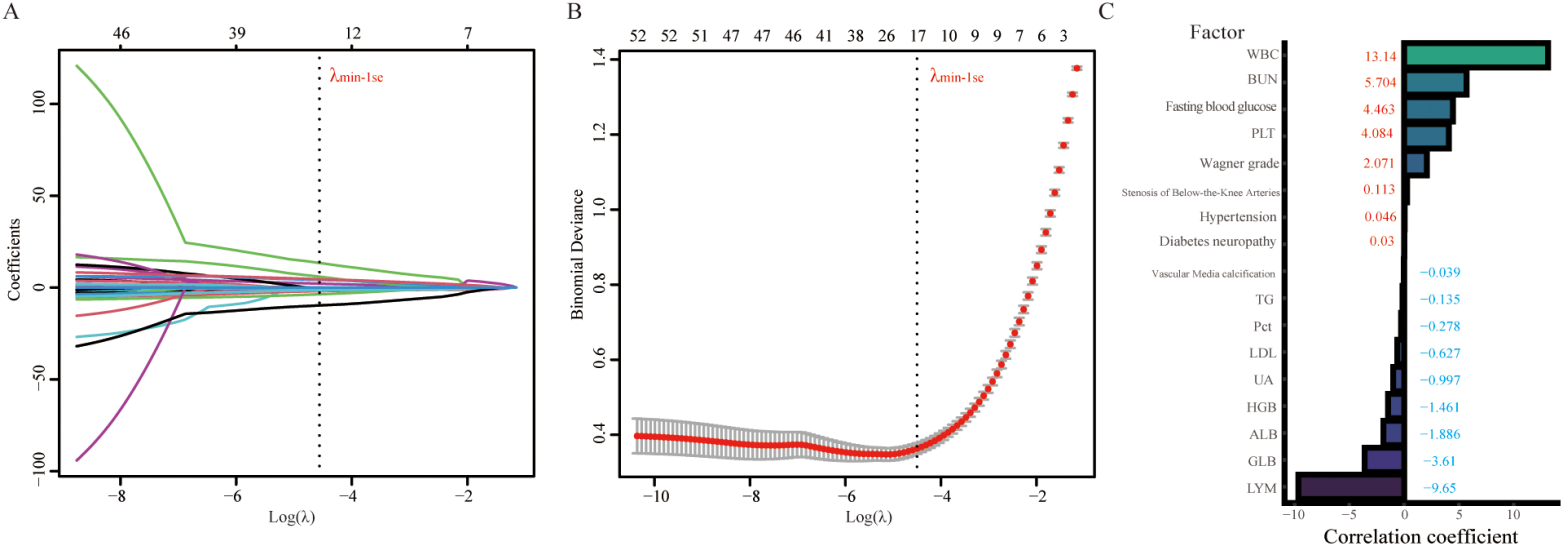
LASSO regression analysis was used to select potential variables. A total of 53 variables were initially included, and 17 variables were ultimately selected for further analysis. **(A)** LASSO coefficient analysis of the clinical features. **(B)** Tuning parameter selection in the LASSO regression model from 10-fold cross-validation. **(C)** Plot of the LASSO coefficient of the 17 candidate predictors for amputation. WBC, white blood cell; BUN, blood urea nitrogen; PLT, platelet; TG, triglyceride; Pct, procalcitonin; LDL, low-density lipoprotein; UA, uric acid; HGB, hemoglobin; ALB, albumin; GLB, globulin; LYM, lymphocyte.

To attain a further reduction in data dimensionality, in the present study, we utilized LASSO regression analysis to identify and select relevant features from the training cohorts. The LASSO regression model incorporated a total of 55 variables, and the plot of the coefficients for this analysis is shown in **Figure 3A**. Each curve represents one variable. For each value of λ, the variables and their corresponding nonzero coefficients form a LASSO model. We subsequently used 10-fold cross-validation to analyze and determine the optimal LASSO regression parameters (28). When λ = 0.0058, the cross-validation error of the model is minimized. Nevertheless, to further reduce the number of variables included in the model, we opted for λmin-1 se (λ = 0.0111) as the final parameter for the LASSO regression analysis (**Figure 3B**) (29). Ultimately, seventeen variables were identified as predictive factors for amputation in the ML model. The regression coefficients of these variables are depicted in **Figure 3C**.

### Model building and evaluation

**Figure 4:**
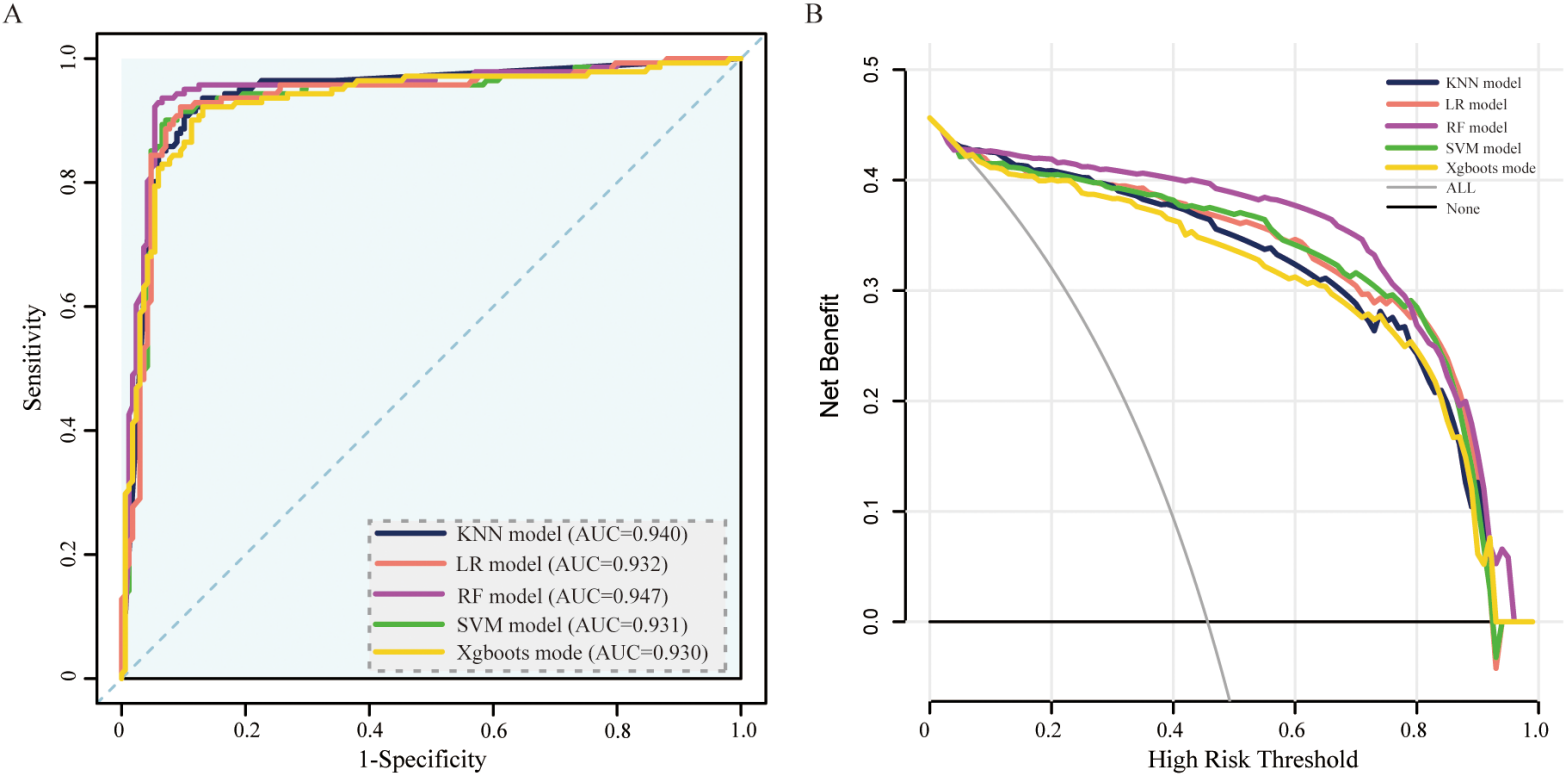
ROC curve and DCA comparison of the 5 models in the internal validation set. **(A)** ROC curves of five ML models for predicting amputation in DFU patients. **(B)** DCA for the 5 models in the internal validation set. AUC, area under the receiver operating characteristic curve; KNN, k-nearest neighbors; LR, logistic regression; SVM, support vector machine; RF, random forest; XGBoost, extreme gradient boosting.

In the model development and validation phase, using GridSearchCV from the sklearn library, we initially identified the optimal hyperparameters for five distinct ML models. Comprehensive details regarding the hyperparameters of the ML models are presented in **Table 2**. The final models were subsequently trained using the optimized hyperparameters and the 17 variables selected through LASSO regression as input features. The five ML models (KNN, LR, SVM, RF, and XGBoost) demonstrated robust discriminative capabilities, as evidenced by their AUCs (95% CI) of 0.94 (0.91-0.97), 0.93 (0.90-0.96), 0.93 (0.90-0.96), 0.95 (0.92-0.98), and 0.93 (0.90-0.96), respectively, in the internal validation set. The AUC curves of the five ML models evaluated on the internal validation dataset are displayed in **Figure 4**. Furthermore, the F1 score was also chosen to compare model performance, as it effectively measures accuracy in imbalanced datasets by harmonizing precision and recall (**Table 2**). Moreover, the DCA is visually presented in **Figure 4B** as an adequate representation of the model’s clinical utility.

**Table 2:** Hyperparameters of the ML models and comparison of performance among the five models in the internal validation cohort.

ML model	Hyperparameters	AUC	Accuracy	Precision	Recall	*F1 score*
KNN	n_neighbors:17; p=1; weights=distance	0.940	0.900	0.915	0.899	0.907
LR	Penalty= l2; C = 1; max_iter:50; solver=liblinear	0.932	0.913	0.932	0.905	0.918
SVM	C=1; gamma=0.01; kernel=linear	0.932	0.948	0.932	0.976	0.953
RF	max_depth=10; min_samples_split=1, min_samples_leaf=1; n_estimators=200; max_features=log2	0.947	0.945	0.931	0.970	0.950
XGBoost	learning_rate = 0.1, n_estimators = 200, max_depth = 2, subsample = 0.8, colsample_bytree = 0.8, gamma = 0.1, objective = ‘binary: logistic’	0.930	0.935	0.957	0.922	0.939

AUC, area under the receiver operating characteristic curve; KNN, k-nearest neighbors; LR, logistic regression; SVM, support vector machine; RF, random forest; XGBoost, extreme gradient boosting.

**Figure 5:**
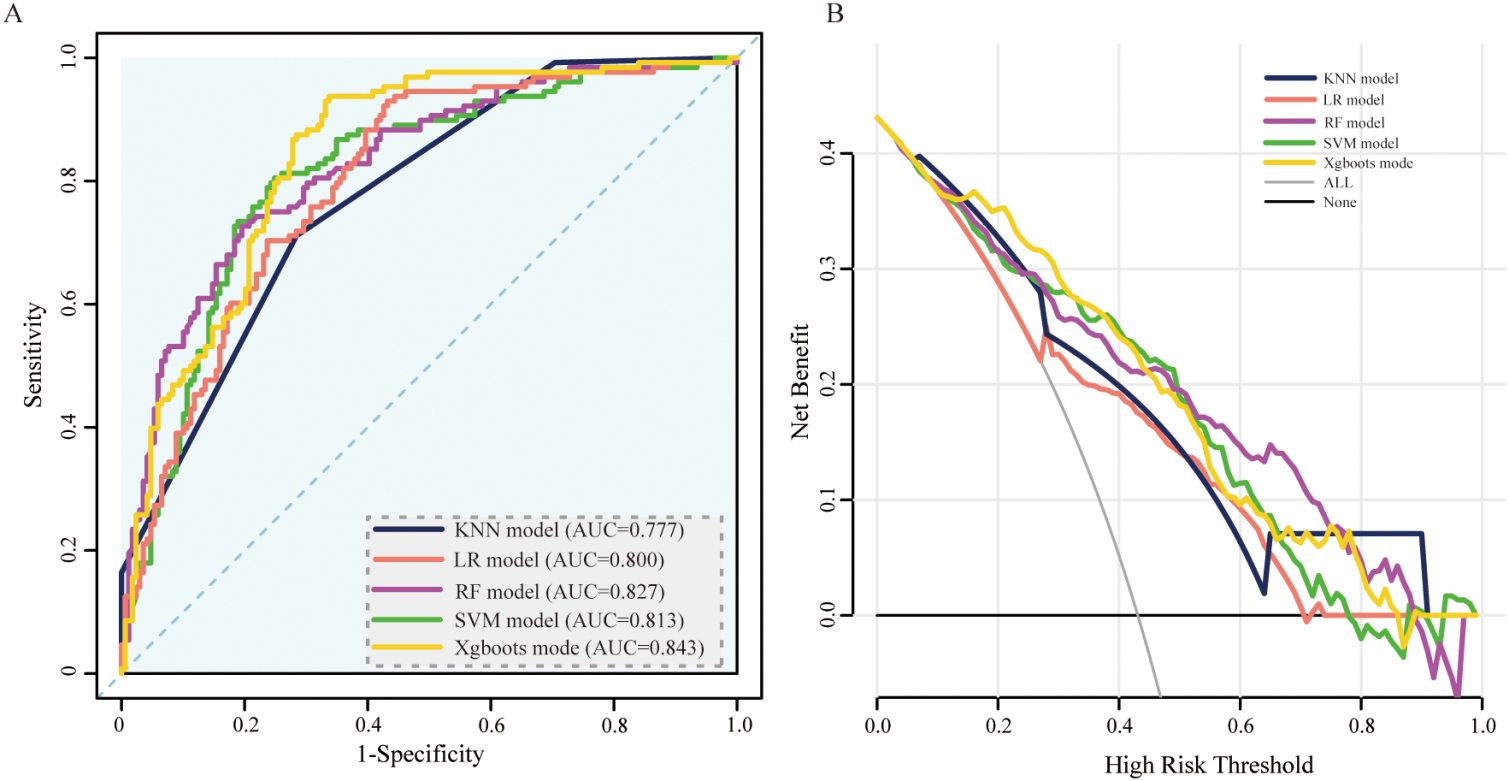
ROC curve and DCA comparison of the 5 models in the external validation set. **(A)** ROC curves of 5 ML models for predicting amputation in DFU patients within the external validation set. **(B)** DCA for the 5 models in the external validation set. AUC, area under the receiver operating characteristic curve; KNN, k-nearest neighbors; LR, logistic regression; SVM, support vector machine; RF, random forest; XGBoost, extreme gradient boosting.

To validate the model in different patients, we created an external validation cohort and rigorously assessed the model’s performance. The external cohort was recruited from four hospitals from 2020–2023. The baseline characteristics of the 17 variables selected as input features for the ML models in the external validation cohorts are detailed in Additional File 1. In the external validation cohort, the AUC values (95% CIs) for the KNN, LR, SVM, RF, and XGBoost models were 0.77 (0.73, 0.82), 0.80 (0.75, 0.85), 0.81 (0.76, 0.86), 0.83 (0.78, 0.87), and 0.84 (0.80, 0.90), respectively (**Figure 5A**). In **Table 3**, we presented a summary of the performance metrics for the five models, including the AUC, accuracy, precision, recall, and F1 score. In addition, **Figure 5B** visually presented the DCA in the external validation cohort, effectively illustrating the model’s clinical utility.

**Table 3:** Comparison of the performance of the five machine learning models within the external validation cohort.

ML model	AUC	Accuracy	Precision	Recall	F1 score
KNN	0.777	0.713	0.766	0.715	0.740
LR	0.800	0.568	0.620	0.930	0.744
SVM	0.813	0.774	0.836	0.751	0.791
RF	0.827	0.768	0.794	0.799	0.796
XGBoost	0.843	0.781	0.933	0.662	0.775

AUC, area under the receiver operating characteristic curve; KNN, k-nearest neighbors; LR, logistic regression; SVM, support vector machine; RF, random forest; XGBoost, extreme gradient boosting.

Compared with the other models, XGBoost consistently demonstrated superior AUCs and F1 scores when evaluated with external validation cohorts. Furthermore, it maintained a high recall rate in the external validation set, thereby demonstrating the model’s ability to accurately identify patients at risk of amputation among those suffering from DFUs. Consequently, subsequent analysis was conducted via the XGBoost model.

### Model interpretability analysis

**Figure 6:**
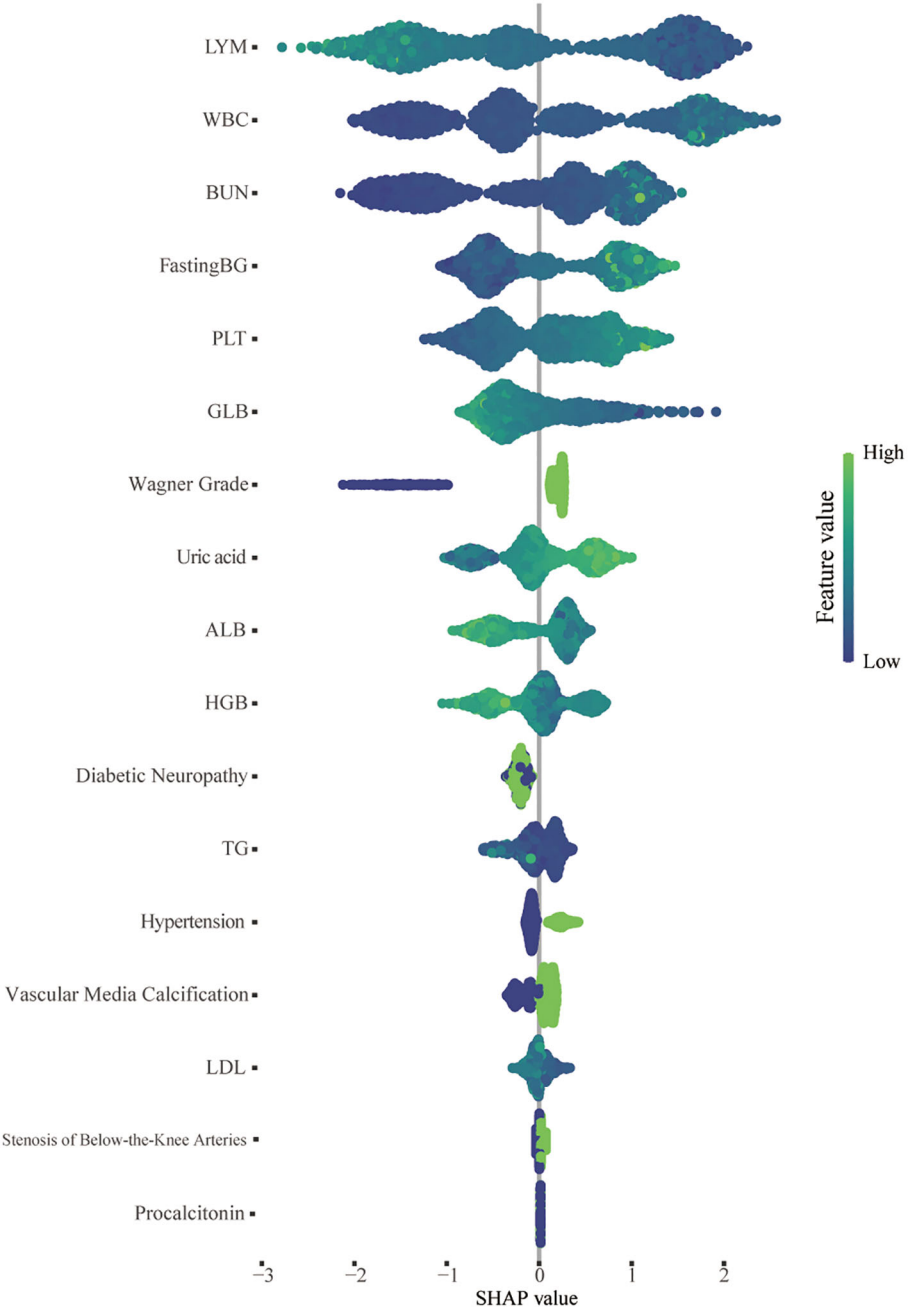
SHAP summary plot of the 17 features of the XGBoost model. For each patient, a dot is generated corresponding to the attribution value of each feature in the model, resulting in one dot per feature per patient on the line. Each line represents a feature, and the abscissa is the SHAP value. The higher the SHAP value of a feature is, the greater the probability of an amputation event. The dots are colored according to the patient’s feature values and are accumulated vertically to describe the density. Green represents a high feature value (in this case, death), whereas blue represents a low feature value. WBC, white blood cell; BUN, blood urea nitrogen; PLT, platelet; TG, triglyceride; Pct, procalcitonin; LDL, low-density lipoprotein; UA, uric acid; HGB, hemoglobin; ALB, albumin; GLB, globulin; LYM, lymphocyte.

The SHAP algorithm was used to assess the importance of each predictive feature in relation to the amputation results, thereby providing further insight into the prediction mechanism used by the XGBoost model. Using SHAP, we assessed the global importance of all 17 features across the entire dataset of training cohorts to elucidate their overall impacts. The results of this analysis are depicted in a summary plot in **Figure 6**. In the SHAP summary plot, positive Shapley values for each feature signify an elevated risk of amputation, whereas negative values denote a diminished risk of amputation. Correspondingly, the colors depicted in the figure represent the magnitude of feature values, with red indicating high feature values and blue denoting low feature values. For example, patients with poor nutritional status (characterized by decreased levels of ALB, LDL, GLB, and TGs) during treatment are more likely to require amputation than those with elevated levels of these biomarkers. Additionally, patients with elevated white blood cell counts or decreased lymphocyte and Hb levels during treatment have a greater risk of amputation than do those without these hematological abnormalities.

**Figure 7:**
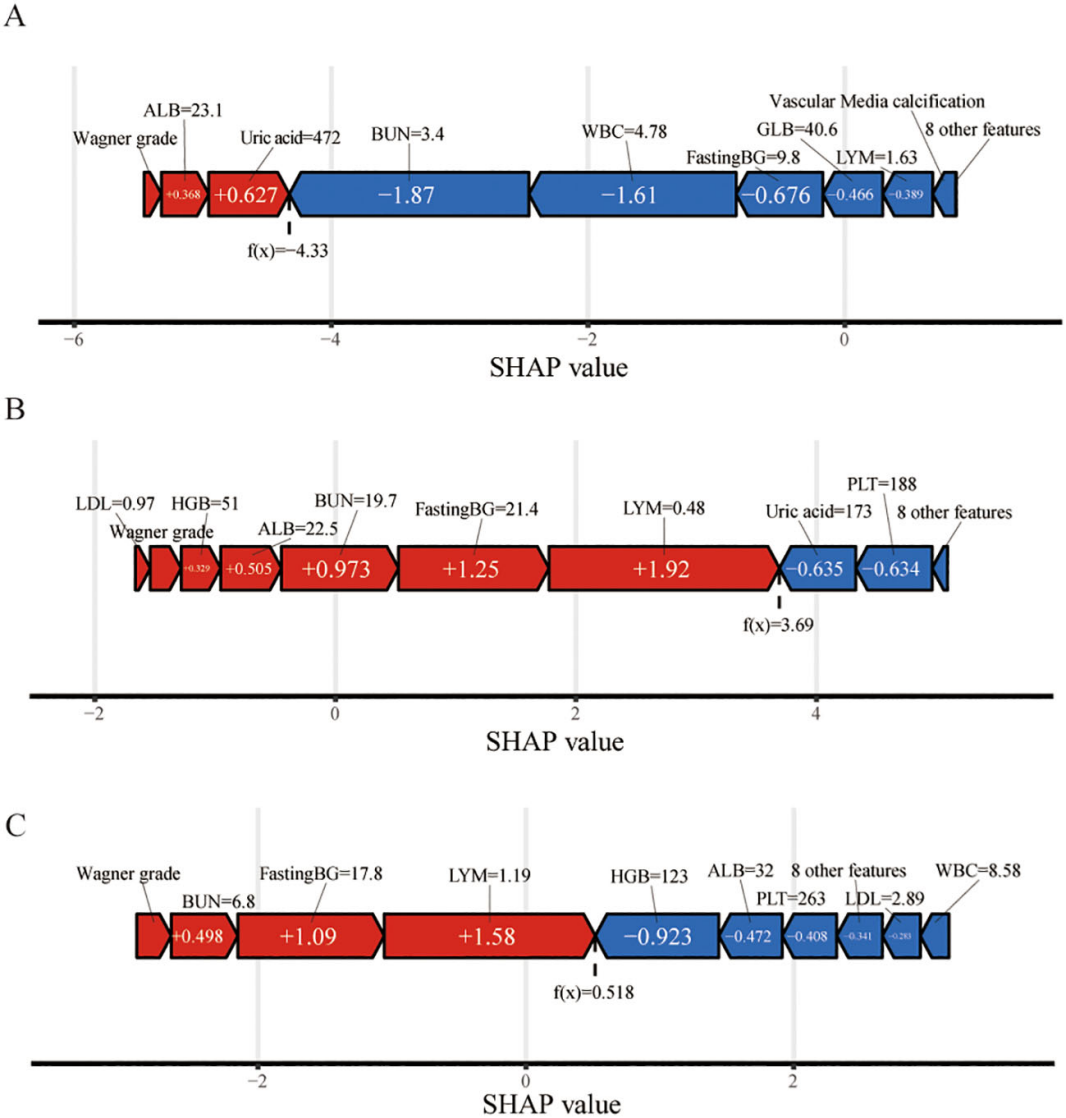
Force plot of model prediction results suggested for three randomly selected samples via SHAP values. The f(x) value represents the output values. The feature values are listed at the top of the plot with feature names. Each group of features was ranked from the center to both ends according to the extent of their impact. The length of the bar for each feature reflects the weight of that feature in the prediction. Factors that increased the predicted score are colored red, and those that decreased the predicted score are shown in blue. **(A)** A patient who did not undergo amputation; **(B)** A patient who underwent amputation; **(C)** Presentation of a patient without amputation via the SHAP method. WBC, white blood cell; BUN, blood urea nitrogen; PLT, platelet; FastingBG, fasting blood glucose; Pct, procalcitonin; LDL, low-density lipoprotein; UA, uric acid; HGB, hemoglobin; ALB, albumin; GLB, globulin; LYM, lymphocyte.

In **Figure 7**, we demonstrate the application of the SHAP method in elucidating individual model predictions, providing an intuitive framework for guiding clinicians’ decision-making processes and deepening their understanding of the model’s predictive mechanisms. The force plots begin with the average of all predictions as their base value. Each predictor, along with its corresponding Shapley value, is depicted by an arrow that either increases (indicated in red) or decreases (indicated in blue) the model’s predicted value. The feature values are listed at the top of the plot. Finally, the convergence points of the red and blue arrows represent the predicted output values of the model. In **Figure 7A**, the patient was diagnosed with a Wagner stage-III DFU. The patient’s lymphocyte count (1.63×10^9^/L), WBC count (4.78×10^9^/L), BUN level (3.4 mmol/L), GLB level (40.6 g/L), and fasting blood glucose level (9.7 mmol/L) were critical parameters for accurately predicting the likelihood of avoiding amputation. However, the patient’s ALB level of 21.6 g/L and Wagner grade were inversely correlated with the prediction of amputation. On the basis of the prediction model, the outcome depicted in **Figure 7A**, where f(x) = −4.33, suggests a high probability of nonamputation. In contrast, the outcomes presented in **Figures 7B, C**, with f(x) values of 3.69 and 0.518, respectively, indicate a relatively high likelihood of amputation.

## Discussion

The aim of this study was to develop and validate five ML models for prognostic prediction in patients with DFUs undergoing amputation, thereby providing clinicians with reliable diagnostic information and treatment options. Ultimately, the XGBoost model was used as the baseline model for the study. The model exhibited exceptional predictive performance across both the internal and the external validation datasets, achieving AUC values of 0.93 and 0.84, respectively. Moreover, with the use of SHAP values and corresponding visualizations, we elucidated the influence of each clinical feature on the performance of the overall XGBoost model. The illustration of feature importance contributes to a comprehensive understanding of the models used for predicting amputation in patients with DFUs.

In the era of big data, a growing array of ML algorithms has been increasingly applied in research on disease risk and prognosis. Previous studies have also conducted assessments of amputation risk for DFU patients. In a prior investigation involving retrospective data from a cohort of 618 patients diagnosed with DFUs, investigators used 37 clinical features to construct an ML model designed to predict the likelihood of amputation in hospitalized patients (18). The final model demonstrated high predictive performance, with an AUC of 0.90. However, the initial dataset used in that study comprised a relatively small sample size, with only 117 amputee patients. Additionally, a considerable number of features were incorporated into the model. These factors may have contributed to overfitting of the model, potentially leading to inaccurate results and undermining the generalizability of the findings. Wang et al. used ML to predict the outcomes of minor amputations in patients with severe wounds (Texas University grade 3+), achieving an AUC of 0.881 (17). Owing to the limited number of amputee cases in the initial dataset, they utilized the synthetic minority oversampling technique (SMOTE) to perform oversampling. SMOTE is used to mitigate data imbalance; however, its use carries the potential risk of inducing model overfitting (30). In contrast to the previously cited studies, our hospital serves as a tertiary referral center, attracting patients with advanced-stage DFUs. This resulted in a more comprehensive and balanced dataset during the initial data collection phase. As previously mentioned, the AUC for the XGBoost model reached values of 0.93 in the internal validation cohorts and 0.83 in the external validation cohorts, demonstrating the strong predictive ability of the model. Nevertheless, the discrepancy in AUC between internal and external validation indicates potential variations in data distribution. The data presented in **Table 1** and **Supplementary Table 1** demonstrate that patients within the training cohorts present a greater prevalence of Wagner grades IV-V. Furthermore, amputee patients in these cohorts are distinguished by advanced age, increased white blood cell counts, and compromised nutritional status. Collectively, these findings indicate a heightened severity of illness among patients in the training cohorts. These differences impact model performance in external validation cohorts and may reduce accuracy for patients with milder symptoms. It is imperative to refine and augment the model by utilizing a larger, multicenter dataset that encompasses patients exhibiting varying severities of DFUs.

Moreover, a notable advantage of our research was the incorporation of the XGBoost algorithm, which has garnered significant attention in recent years owing to its rapid computational efficiency, robust generalization properties, and superior predictive performance (31–33). Furthermore, we used GridSearchCV for the optimization of hyperparameters. In our analysis, the p value for the difference in the AUC between XGBoost and the other models was not statistically significant. Nonetheless, it is crucial to underscore that the selection of an optimal model transcends mere statistical significance. Unlike logistic regression (linear) or SVM (kernel-dependent), XGBoost automatically captures complex feature interactions through sequential tree-building., which is vital for patients with DFUs (34). Each new tree corrects residuals from previous trees, modeling intricate patterns that linear models or single decision trees miss. XGBoost incorporates L1 and L2 regularization directly into its objective function, penalizing overly complex trees. This reduces overfitting, a critical weakness of KNN. Moreover, unlike RF (which builds trees independently), XGBoost uses gradient boosting to improve predictions iteratively. This error-correction mechanism allows it to refine model performance more effectively. Parameters such as max_depth, learning_rate, and subsample allow fine-grained control over bias-variance tradeoffs. While RF requires the tuning of fewer parameters, it lacks adaptability to the boosting framework. Additionally, assessing metrics such as recall, accuracy, and the F1 score further substantiates the robust predictive performance of the XGBoost model.

An additional strength of our study is the application of SHAP for the interpretation of the XGBoost model, which helped with the identification of important variables linked to amputation risk. In the final model, the WBC count and lymphocyte count were the most significant features for model selection, given that these parameters are frequently associated with systemic inflammatory responses and the severity of infection in patients (35). A series of infection markers (such as the WBC count, CRP level and erythrocyte sedimentation rate) have long been regarded as predictive indicators for LEA in patients with DFUs (36, 37). This observation is consistent with clinical experience, indicating that a pronounced inflammatory response exacerbates tissue damage, impedes reparative mechanisms, and significantly elevates the risk of amputation. Moreover, nutritional status is also a predictor of amputation. In our study, nonamputee patients presented elevated levels of serum ALB, GLB, and Hb, which serve as indicators of patients’ nutritional status. In alignment with these findings, a study of 3,654 DFU patients revealed that lower Hb and plasma ALB levels independently increase the risk of amputation (38). Interestingly, although an adverse lipid profile or dyslipidemia is a significant risk factor for various diabetic complications (39–41), our study indicates that reduced lipid levels in DFU patients often signify a poor prognosis. Therefore, clinicians should pay close attention to the nutritional status of DFU patients and promptly address issues related to anemia and malnutrition to improve the overall condition of these patients and promote wound healing.

In the present study, predictors of amputation in patients with DFUs were examined and found to align with previous research. Specifically, our findings corroborate earlier studies indicating that elevated BUN levels are positively correlated with higher rates of amputation (42). BUN serves as a biomarker indicative of renal function in patients. Increased BUN levels are frequently correlated with compromised kidney function (43). Compromised renal function can result in edema and metabolic disturbances in patients, hindering the healing process of DFU and culminating in amputation. Moreover, elevated fasting blood glucose levels, stenosis of below-the-knee arteries, and increased uric acid levels are significantly correlated with poor prognosis in DFU patients (44–46). The incorporation of a diverse range of features has enabled our model to achieve favorable predictive performance, resulting in strong efficacy and generalizability across both internal and external validation cohorts.

In the future, the implementation of our XGBoost model in clinical settings offers transformative potential for developing precise management strategies for patients with DFUs. It possesses the ability to perform real-time analysis of patient data as they are collected, providing clinicians with immediate risk stratification and predictive insights. For example, during patient admissions, the model can evaluate risk factors for conditions of DFUs, thereby facilitating timely interventions. In the future, the integration of our XGBoost models as plugins or via APIs into electronic health record (EHR) systems is anticipated. This integration will facilitate clinicians’ access to predictive analytics at the commencement of treatment. This could allow for better population-based strategies to identify amputations and more precisely target prevention or treatment resources to patients who would benefit the most.

Nevertheless, our study is subject to certain limitations. The development of the predictive model is restricted primarily by the features used during its training. There may be additional features that serve as useful predictive factors for DF amputation risk that were not identified in this study. For instance, the lack of assessment to classify patients’ DFUs into neuropathic, ischemic, and neuro-ischemic categories upon admission has resulted in insufficient data on the types of DFUs. The omission of these factors could introduce potential bias. Second, the retrospective design of this study inherently leads to instances of missing data, constituting another limitation of our research. Further validation through prospective studies is warranted. Third, the features utilized for modeling in this study were exclusively gathered from patients during admission. Should these features be collected at multiple time points and across various care settings, the resulting dataset would be more comprehensive. Fourth, our hospital is designated as a tertiary care facility in Guangdong Province, which frequently results in the referral of patients with severe DFUs through multiple channels. As a result, the patients managed at our institution typically present with more advanced conditions and an increased likelihood of requiring amputation. These differences may affect the accuracy of models when they are applied to patients exhibiting milder symptoms. It is essential to incorporate a larger multicenter dataset. Fifth, due to the limited number of major amputation cases, we did not assess the risks of major and minor amputations in patients with diabetic foot ulcers separately. In the future, it is crucial to gather more patient data to analyze the disparate risk factors for major and minor amputations comprehensively. This will facilitate enhanced risk prediction for both types of amputation.

## Conclusion

In conclusion, ML models have emerged as reliable tools for amputation prediction in patients with DFUs. The adoption of explainable modeling techniques, such as SHAP, offers insights into the significance of individual features in contributing to the model’s output, thereby enhancing the transparency and feasibility of model deployment. Therefore, the model can serve as a valuable reference for clinicians in tailoring precise management strategies for patients with DFUs. With additional prospective validation and refinement, this model has the potential to identify patients at high risk for amputation, thereby contributing to a reduction in the overall amputation rate in DFU patients. Future research could incorporate additional novel biomarkers and prospective data to refine and enhance the prediction model, rendering it more comprehensive and holistic.

## Data Availability

The raw data supporting the conclusions of this article will be made available by the authors, without undue reservation.
