# Supplementary material for: An interpreting machine learning models to predict amputation risk in patients with diabetic foot ulcers: a multi-center study
Source: Front Endocrinol (Lausanne). 2025 Mar 25;16:1526098. doi: 10.3389/fendo.2025.1526098 (PMC11975565; doi:10.3389/fendo.2025.1526098)
Supplement: Supplementary file 1 [file Table1.doc]

Supplement table.1 The characteristics of external validation set patients when first admission

| Factors | Total  (n=297) | Non-Amputation  (n=169) | Amputation  (n=128) | *P* values |
| --- | --- | --- | --- | --- |
| **Demographics** |  |  |  |  |
| Age, yr | 66.1±12.2 | 66.9±12.4 | 64.9±11.8 | 0.102 |
| Sex, n (%) |  |  |  | 0.945 |
| Male | 119(40.1%) | 68(45.0%) | 51 (41.4%) |  |
| Female | 178(59.9%) | 101(55.0%) | 77(58.6%) |  |
| Duration of diabetes, yr | 10.0 (5.0-19.0) | 10.0 (6.0-20.0) | 10.0 (4.0-15.0) | 0.155 |
| **Medical history** |  |  |  |  |
| Hypertension, n (%) | 168(56.6%) | 93(55.0%) | 75(58.6%) | 0.539 |
| Diabetic peripheral neuropathy, n (%) | 224(75.4%) | 121(71.6%) | 103(80.5%) | 0.079 |
| **Wagner classification system, n (%)** |  |  |  | <0.001 |
| I-III | 112(37.7%) | 106(62.7%) | 6(4.7%) |  |
| IV-V | 185(62.3%) | 63(37.3%) | 122(95.3%) |  |
| **Clinical and laboratory data** |  |  |  |  |
| WBC count, ×10^9^/L | 11.2±5.6 | 10.1±5.0 | 12.6±6.0 | <0.001 |
| Hemoglobin, g/L | 114.0±22.4 | 117.3±22.8 | 109.7±.21.2 | 0.004 |
| PLT count, ×10^9^/L | 281(75-929) | 263(218-340) | 302(236-362) | 0.012 |
| LYM count, ×10^9^/L | 1.64±0.82 | 1.4(1.1-1.8) | 1.4(1.0-1.8) | 0.645 |
| Globulin, g/L | 32.8±6.5 | 34.4±6.5 | 31.6±6.2 | <0.001 |
| Albumin, g/L | 33.7±6.3 | 34.5±6.1 | 32.6±6.5 | 0.013 |
| BUN, mmol/L | 8.7±5.5 | 8.7±5.3 | 8.7±5.5 | <0.001 |
| Uric acid, μmol/L | 347 (102-735) | 358(116-713) | 327(102-735) | 0.076 |
| Triglycerides, mmol/L | 1.4±0.9 | 1.49±0.9 | 1.4±0.8 | 0.785 |
| LDL, mmol/L | 2.7±1.0 | 2.7±1.0 | 2.7±0.9 | 0.853 |
| Fasting blood glucose, mmol/L | 11.76±5.21 | 9.4±3.3 | 11.8±5.7 | <0.001 |
| Procalcitonin, ng/ml |  |  |  | <0.001 |
| ≤0.5, n (%) | 150(50.5%) | 86(50.9%) | 64(50.0%) |  |
| ＞0.5, n (%) | 147(49.5%) | 83(49.1%) | 64(50.0%) |  |
| **Lower limb vascular imaging examination** |  |  |  |  |
| Vascular Media calcification, n (%) | 133(44.8%) | 72(42.6%) | 61(47.7%) | 0.386 |
| Stenosis of Below-the-Knee Arteries, n (%) | 143(48.1%) | 66(39.1%) | 77(60.2%) | <0.001 |

Mean ± SD(standard deviation)for continuous variables with normal distributions, and the p value was calculated by with independent samples t tests. Non-normally distributed variables are expressed as the median (inter-quartile ranges),and comparisons were conducted using the Kruskal-Wallis test. Categorical data were showed as frequency (percentage)

Abbreviations: WBC, white blood cell; PLT, platelet; LYM, lymphocyte; GLB, globulin; ALB, albumin; BUN, blood urea nitrogen; LDL, low-density lipoprotein.
